# Antimicrobial-Resistant *Escherichia coli* Survived in Dust Samples for More than 20 Years

**DOI:** 10.3389/fmicb.2016.00866

**Published:** 2016-06-10

**Authors:** Jochen Schulz, Inga Ruddat, Jörg Hartung, Gerd Hamscher, Nicole Kemper, Christa Ewers

**Affiliations:** ^1^Institute for Animal Hygiene, Animal Welfare and Farm Animal Behaviour, University of Veterinary Medicine Hannover, FoundationHannover, Germany; ^2^Department of Biometry, Epidemiology and Information Processing, University of Veterinary Medicine Hannover, FoundationHannover, Germany; ^3^Institute of Food Chemistry and Food Biotechnology, Justus Liebig University GiessenGiessen, Germany; ^4^Institute of Hygiene and Infectious Diseases of Animals, Justus Liebig University GiessenGiessen, Germany

**Keywords:** survival, *Escherichia coli*, dust, livestock, antibiotic, resistance, fluoroquinolones, ciprofloxacin

## Abstract

In a retrospective study, 119 sedimentation dust samples stored between five and 35 years from various barns of intensive livestock farming were evaluated for the occurrence of cultivatable *Escherichia coli*. Growth of *E. coli* occurred in 54 samples. Successful cultivation was achieved in samples from as early as 1994. The frequency of detection increased from earlier to later time periods, but the concentrations, which ranged between 3.4 × 10^2^ and 1.1 × 10^5^ colony-forming units per gram, did not correlate with sample age (Spearman rank correlation; *p* > 0.05). We hypothesize that *E. coli* cells survived in dust samples without cell division because of the storage conditions. Dry material (dust) with low water activities (arithmetic mean < 0.6) and storage at 4°C in the dark likely facilitated long-term survival. *E. coli* were isolated on MacConkey agar with and without ciprofloxacin supplementation. For 110 isolates (79 from non-supplemented media and 31 from supplemented media), we determined the *E. coli* phylotype and antimicrobial resistance. Six phylogenetic groups were identified. Phylogroups A and B1 predominated. Compared to group A, phylogroup B1 was significantly associated with growth on ciprofloxacin-supplemented media (chi-square test, *p* = 0.003). Furthermore, the antibiotic resistance profiles determined by a microdilution method revealed that isolates were phenotypically resistant to at least one antimicrobial substance and that more than 50% were resistant to a minimum of five out of 10 antibiotics tested. A linear mixed model was used to identify factors associated with the number of phenotypic resistances of individual isolates. Younger isolates and isolates from fattening poultry barns tended to be resistant to significantly more antibiotics than older isolates and those from laying-hen houses (*p* = 0.01 and *p* = 0.02, respectively). Sample origin and storage conditions may have influenced the number of antimicrobial resistances. Overall, we found that under particular conditions, dust from farm animal houses can be reservoirs for antimicrobial-resistant *E. coli* for at least 20 years. The survival strategies that allow *E. coli* to survive such long periods in environmental samples are not fully understood and could be an interesting research topic for future studies.

## Introduction

Dust in livestock buildings originates mainly from feed, skin, feathers, bedding material, and feces (Carpenter, 1986). Its organic mass fraction is about 85%. The material is commonly dry (water content near 10%) and contains about 25–70% crude protein and 1–8% crude fat (Hartung and Saleh, 2007). Furthermore, it contains a variety of biological particles and compounds which can affect the health of both exposed animals and farm workers (Seedorf and Hartung, 2001; Rylander and Carvalheiro, 2006).

Knowledge about the microbiota of animal house dust originates primarily from air sampling, performed with active air samplers, followed by culture-based methods. Only a few studies used passive sampling. Although the spectra and quantities of the airborne microbiota is very likely influenced by the sampling method and stress factors associated with these methods (Thorne et al., 1992; Stewart et al., 1995; Pahl et al., 1997; Li, 1999), valid observations were generally made in livestock buildings of various farm animals. Independent of sampling method (passive vs. active), the most prevalent microorganisms in dust are Gram-positive cocci (Seedorf et al., 1998; Hartung and Saleh, 2007; Normand et al., 2011). For instance, in animal houses of various species, between 8 × 10^7^ and 8 × 10^9^ colony-forming units (CFU) per gram were detected in the dust, and the *Staphylococcus* spp. ranged between 30 and 83% of the total microorganism count (Hartung and Saleh, 2007). This genus also predominated the airborne bacteria in poultry barns (Friese et al., 2012).

Besides Staphylococci, Streptococci also account for most of the airborne microbiota in pig barns (Hartung and Saleh, 2007; Schulz et al., 2013). Among airborne micrococci in livestock buildings are potential pathogens and antimicrobial-resistant strains. This is also true for the generally less- concentrated *Enterobacteriaceae* bacteria (1–2% of the total bacterial count). For instance, pathogenic and antibiotic-resistant *Escherichia coli* were found in the air of pig and poultry barns (Letourneau et al., 2010; Laube et al., 2014).

The potential transmission of these bacteria to human beings and the contamination of the environment by manure or airborne emissions are critically judged (van den Bogaard et al., 2001; Khanna et al., 2008). Furthermore, *E. coli* isolates from animals and other sources of animal husbandry act as indicator bacteria to monitor the occurrence and persistence of antimicrobial resistance in food animals (van Hoorebeke et al., 2011). Dusts from farm animal houses seem to be potential reservoirs of *E. coli* (Harry, 1964). Analyzing stored samples may offer a look into the past concerning the occurrence of antimicrobial resistance in *E. coli*. For instance, fluoroquinolones were introduced in the late 1980s and early 1990s for food animal use in European countries (Endtz et al., 1991; Engberg et al., 2001). *E. coli* isolates from dust samples prior to and after this introduction may reveal influences of its usage. However, an essential precondition for such a study would be sufficient survival of *E. coli* in animal house dust. To our knowledge, systematic studies investigating the long-term survival of *E. coli* in dust from farm animal houses are missing. Also, studies about the survival of *E. coli* in dry materials are scarce. In a review about the persistence of *E. coli* in the open environment, the authors stated that *E. coli* survived for almost 1 year (van Elsas et al., 2011). Nonetheless, two other published articles yielded valuable information about *E. coli* survival, especially in dry material. In a study by Gundermann (1972) *E. coli* isolates from dust were shown to survive for about 2.5 years at relatively low humidity. Additional important information was supplied by Louis et al. (1994), who observed no decrease in viable *E. coli* K-12 after air drying and storage at 4°C for 26 weeks. Although the authors worked with dried pure cultures, which are not comparable to complex animal house dust, the conditions (4°C and low water availability) were comparable to those of the samples we used, which were stored for up to 35 years after being taken from various livestock buildings. Therefore, a retrospective study was conducted to investigate the long-term survival of *E. coli* in stored dust samples. Isolates from various time periods were characterized by phylotyping and antimicrobial susceptibility testing.

## Materials and Methods

### Origins of Dust Samples

One hundred and nineteen sedimentation dust samples were collected between 1980 and 2009 in five pig and eight poultry barns located in Northern Germany. Sampling dates and origins are listed in **Table 1**. Samples were taken as parts of various studies to quantify the mass of sedimentation dust per area and time. Each sample was taken by allowing airborne dust to settle freely on a rectangular metal sheet (100 cm × 40 cm, upstand 2 cm), which was covered with new sterile aluminum foil at the beginning of each sampling period. Each ready-to-sample metal sheet was positioned in the central area of one of the barns, approximately 1.5 m above the barn floor, out of reach of the animals and of direct influence of incoming air. After about 4 weeks, the aluminum foil was carefully folded in the barn, transferred to a sterile plastic bag, and taken to the laboratory where the weighed dust (between 5 and 50 g) was stored in a sterile glass cylinder subsequently sealed with a sterile cork. Cylinders were stored in an air-conditioned room at the Institute for Animal Hygiene, Animal Welfare and Farm Animal Behavior in Hannover, Germany, at 4°C in the dark.

**Table 1:** Origins of dust samples and sampling times.

Barn	Housed animals	(Sample no) and sampling date
1	Pigs	(1) January 1980, (2) March 1981, (3) July 1982, (4) August 1983, (5) August 1984, (6) August 1985, (7) August 1986, (8) August 1987, (9) August 1988, (10) July 1989, (11) September 1990, (12) November 1991, (13) August 1992, (14) August 1993, (15) May 1994, (16) July (1995), (17) July 1996, (18) July 1997, (19) July 1998, (20) August 1999, (21) May 2000
2	Pigs	(22) December 2004, (23) January 2005, (24) February 2005, (25) March 2005, (26) April 2005, (27) May 2005, (28) June 2005, (29) July 2005, (30) August 2005, (31) September 2005, (32) October 2005, (33) November 2005, (34) December 2005
3	Pigs	(35) May 2007, (36) May 2007, (37) March 2009, (38) March 2009, (39) April 2009, (40) April 2009, (41) April 2009, (42) April 2009, (43) May 2009, (44) May 2009, (45) May 2009, (46) June 2009, (47) June 2009, (48) June 2009, (49) June 2009, (50) June 2009
4	Pigs	(51) March 2009, (52) May 2009, (53) June 2009, (54) June 2009, (55) June 2009, (56) June 2009, (57) June (2009)
5	Pigs	(58) January 2000, (59) January 2000, (60) March 2000
6	Broilers	(61) August 2003, (62) August 2003, (63) September 2003, (64) Set. 2003, (65) November 2003, (66) November 2003
7	Broilers	(67) February 2004, (68) February 2004, (69) February 2004, (70) May 2004, (71) June 2004, (72) December 2004, (73) December 2004, (74) December 2004, (75) January 2005, (76) January 2005, (77) January 2005, (78) January 2005, (79) January 2005, (80) March 2005, (81) March 2005, (82) April 2005, (83) April 2005, (84) June 2005, (85) July 2005, (86) September 2005, (87) December 2005, (88) December 2005
8	Laying hens	(89) April 2005, (90) April 2005, (91) April 2005, (92) June 2005, (93) June 2005, (94) June 2005, (95) November 2005, (96) November 2005, (97) November 2005
9	Laying hens	(98) March 2009
10	Broilers	(99) June 1994, (100) July 1994, (101) August 1994, (102) September 1994, (103) October 1994, (106) June 1992, (107) July 1992, (108) December 1992, (109) February 1994, (110) October 1994
11	Broilers	(104) March 1992, (105) October 1993
12	Ducks	(111) September 2003, (112) March 2004, (113) January 2005, (114) November 2005
13	Turkeys	(115) January 2004, (116) May 2004, (117) July 2004, (118) December 2004, (119) October 2005

### Water Activities of Dust Samples

The water activity was measured by the Aquaspector AQS-31 (NAGY Messsysteme GmbH, Gäufelden, Germany). Measurements were performed by means of the operating instructions of the manufacturer. Thirty-six samples from pig barns and 42 samples from poultry houses were analyzed immediately before preparing the samples for microbiological analyses. Forty-one samples contained too little dust to measure the water activity.

### Isolation and Quantification of *E. coli* and Total Viable Count of Mesophilic Bacteria

Isolation and quantification of *E. coli* and total viable bacteria counts were determined between 09/09/2014 and 01/06/2015. To isolate and to quantify *E*. coli, 0.1 g dust was dissolved in 10 ml PBS buffer + 0.01% TWEEN20 (v/v). The suspension was shaken for 30 min in a water bath at 25°C. Subsequently, the suspension was vortexed for 4 min at Stage 6 with a VORTEX-2 GENIE^TM^ (Scientific Industries Inc., Bohemia, NY, USA). Aliquots (0.5 ml, 0.1 ml, and 0.1 ml of a 10-fold dilution) were plated in triplicate on MacConkey agar (Oxoid Ltd., Basingstoke, UK) and also on MacConkey agar supplemented with 2 μg/ml ciprofloxacin (CIP; Sigma–Aldrich Chemie GmbH, Steinheim, Germany) to prescreen for *E. coli* isolates with reduced susceptibility to CIP (Liss et al., 2013; CLSI, 2014). Plates were incubated at 36°C for 48 h. Presumed *E. coli* colonies were counted, and the number of *E. coli* per gram dust was calculated. Negative controls were prepared by inoculating supplemented (MacCCIP) and non-supplemented (MacC) MacConkey media with 0.5 ml buffer. As a positive control, a CIP-resistant *E. coli* isolate (no. 514/1/16, Institute for Microbiology, University of Veterinary Medicine Hannover, Foundation, Germany) was streaked out on MacConkey agar with and without CIP. Controls were incubated simultaneously with the samples. Furthermore, the total viable count of aerobic mesophilic bacteria in dust was examined by plating a dilution series from the same dust suspension used for *E. coli* isolation. Viable counting was carried out on Tryptone Soya Agar plates (Oxoid Ltd, Basingstoke, UK). Plates were incubated at the same time and under the same conditions as MacConkey media. Samples collected over 30-year period were divided by sample date into 5-year sections for the purposes of describing results.

### Identification and Phylotyping

Overall, 113 presumed *E. coli* colonies obtained from 54 dust samples were streaked out on Columbia Agar with sheep blood (PB5008A; Oxoid Deutschland GmbH, Wesel, Germany). Plates were incubated at 36°C for 24 h. One single colony from each plate was used to prepare a suspension to inoculate API^®^ 20 E biochemical test strips according to manufacturers protocol (bioMérieux SA, Marcy-l’Étoile, France). Results were analyzed by means of the apiweb^TM^ - API 20 E V4.1 software (licensed by bioMérieux, Deutschland GmbH, Germany). Isolates significantly identified as *E. coli* were assigned to one of the six phylogroups (A, B1, B2, D, C, and F) based on the method published by Clermont et al. (2013). Briefly, bacterial DNA of the *E. coli* isolates was extracted by using the Master Pure^TM^ Genomic DNA Purification Kit for blood version II (Biozym Diagnostic GmbH, Germany), and DNA was diluted to a working concentration of 50 ng/μl. The PCR reaction mixture contained 5 μl of the DNA template, 4.5 μl 10x Dream Taq Green Buffer, 0.15 μl Green Taq Polymerase (5 U/μl; both Thermo Fisher Scientific Inc., Dreieich, Germany), 0.5 μl 0.2 mM dNTPs (PAN-Biotech GmbH, Aidenbach, Germany), 16.5 μl A. bidest., and 0.5 μl each of the published forward and reverse primers (10 pmol/μl stock solution). Cycling conditions were used as previously reported (Clermont et al., 2013), and the PCR reaction was performed in a TProfessional Trio Thermocycler (Biometra GmbH, Göttingen, Germany). The number of isolates typed per sample and their origins are shown in Supplementary Table 1.

### Antimicrobial Susceptibility Testing

The resistant phenotype of each typed isolate was determined using a microdilution test with the VIZION^®^ system (TREK Diagnostik Systems Ltd., West Sussex, UK). Plates (plate code CMV3AGNF) were inoculated with *E. coli* suspensions as proposed by the manufacturer’s protocol. Antibiotic resistance to amoxicillin/clavulanic acid (AUG2), ampicillin (AMP), cefoxitin (FOX), ceftriaxone (AXO), chloramphenicol (CHL), CIP, gentamicin (GEN), sulfisoxazole (FIS), tetracycline (TET), and trimethoprim/sulfamethoxazole (SXT) were analyzed by means of the Sensititre^TM^ SWIN^TM^ Sofware (p/N SW514-2, February 27, 2014) adjusted to use Clinical and Laboratory Standards Institute (CLSI) breakpoints (CLSI, 2014). If an invalid result for at least one of the tested drugs (e.g., if it contained multiple skips) was obtained, the test was repeated. This occurred in 11 cases.

### Statistical Analyses

Statistical analyses were performed using SAS 9.3 (SAS Institute Inc., Cary, NC, USA). A linear mixed model was used to analyze factors that had possible influences on the number of phenotypic resistances (dependent variable) of *E. coli* isolates. Overall, 103 typed isolates (49 were assigned to phylogenetic group A, 48 to group B1, and 6 to group D) were included in the model. Seven of 110 identified isolates (representing phylogroups B2, C, and E) were not considered because their frequencies were insufficient. The fixed effects were chosen to be the age of isolates in months, animal group (fattening poultry, laying hens, or pigs) from which the isolates originated, the medium (MacC or MacCCIP) used for *E. coli* isolation, and assigned phylogroup. The random effect was chosen to be the barn sampled. Data used to parameterize the model are shown in Supplementary Table 1. We recognized statistical significance when *p* ≤ 0.05. If we found significance with more than two groups for a factor, we conducted pairwise comparisons between groups using differences of least squares means.

## Results

### Water Activities of Dust Samples

Water activity (*a*_w_) was determined for 36 samples from pig barns and 42 samples from poultry barns. In pig barn dusts, the average (standard deviation [SD]) *a*_w_ was 0.567 (0.035), and in poultry barn dust, it was 0.599 (0.038).

### Growth Controls

**FIGURE 1:**
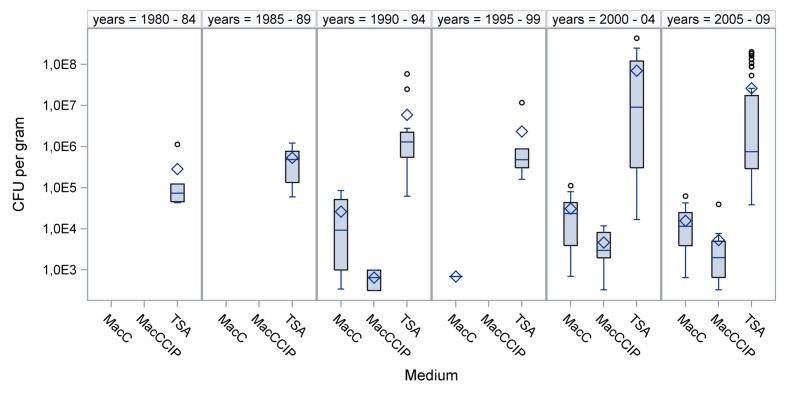
**Bacterial concentrations in dusts collected during various time periods.** Total viable bacteria were counted on TSA agar, and Escherichia coli were counted on MacConkey agar (MacC) and on MacConkey agar with ciprofloxacin (MacCCIP). ◊ = arithmetic mean, ○ = outlier, – = median

All dust samples (*N* = 119) showed growth of aerobic mesophilic bacteria. The total viable counts on TSA agar varied between 1.7 × 10^4^ and 4.3 × 10^8^ CFU/g dust. The arithmetic mean (SD) was 2.9 × 10^7^ (6.7 × 10^7^) CFU/g dust. Medians are given in **Figure 1**. Notably, the number of total bacteria did not correlate with sample age (Spearmans correlation coefficient = -0.019; *p* = 0.836). No bacterial growth occurred on MacConkey agar inoculated with suspension buffer only (negative controls). Positive controls always showed growth of *E. coli* on MacConkey agar, both with and without CIP.

### Detection and Concentrations of *E. coli*

**Table 2** summarizes the origins of dust samples from the six 5-year time periods by *E. coli* test result (positive or negative). *E. coli* was isolated from 54 of the 119 dust samples. Fifteen samples from a pig barn (barn 1) and two samples from a broiler barn (barn 11), all of which were taken between January 1980 and May 1994, tested negative. The oldest samples in which *E. coli* was successfully cultivated were collected between June and October 1994 (samples 99, 100, 102, and 103 in **Table 1**). Only one pig barn was sampled annually from 1995 to 1999, and *E. coli* was found only in the samples from 1995. Subsequent to this, the number of sampled barns and also the number of samples increased. Simultaneously, the relative frequencies of *E. coli*-positive dust samples increased.

**Table 2:** *Escherichia coli* test results for dust samples collected during six time periods.

Time period	1980–1984	1985–1989	1990–1994	1995–1999	2000–2004	2005–2009
No. of negative samples (barn, housed animals)	5 (barn 1, pigs)	5 (barn 1, pigs)	5 (barn 1, pigs) 5 (barn 10, broilers) 2 (barn 11, broilers)	5 (barn 1, pigs)	1 (barn 2, pigs) 3 (barn 5, pigs) 5 (barn 6, broilers) 1 (barn 12, ducks) 1 (barn 13, turkeys)	1 (barn 2, pigs)14 (barn 3, pigs) 7 (barn 4, pigs) 1 (barn 7, broilers) 1 (barn 9, laying hens) 2 (barn 12, ducks)
No. of positive samples (barn, housed animals)	0	0	4 (barn 10, broilers)	1 (barn 1, pigs)	1 (barn 1, pigs) 1 (barn 6, broilers) 8 (barn 7, broilers) 1 (barn 12, ducks) 3 (barn 13, turkeys)	11 (barn 2, pigs) 2 (barn 3, pigs) 13 (barn 7, broilers) 9 (barn 8, laying hens) 1 (barn 13, turkeys)
Relative frequency of positive samples	0	0	0.25	0.17	0.56	0.58

Overall, samples from poultry barns were more often positive for *E. coli* than samples from pig barns (chi-square test, *p* < 0.001). Forty of 58 samples (68%) from poultry barns were positive, whereas 15 of 61 samples (25%) from pig barns were positive. This might have been because more samples were obtained from pig barns between 1980 and 1999. However, in the latest two time periods, 11 samples from poultry barns and 26 from pig barns were negative, whereas 37 samples from poultry barns and 14 samples from pig barns were positive. A chi-square test based on these numbers indicated significantly more *E. coli*-positive samples from poultry barns (*p* < 0.001).

The concentrations of *E. coli* and total aerobic mesophilic bacteria in specific cultivation media are compared in **Figure 1**. The medians of total viable counts, compared to *E. coli* counts, from MacConkey agar showed about 100-fold higher concentrations. When *E. coli* could be calculated from MacCCIP, the numbers were significantly lower than that found in non-supplemented media. Furthermore, concentrations from both, supplemented and non-supplemented media were non-normally distributed (Kolmogorov–Smirnov test, *p* < 0.01). No correlations were observed between the concentrations and sample age (Spearman correlation coefficients 0.0839 and 0.0808, respectively; *p* > 0.05).

### *Escherichia coli* Identification and Phylotyping

One hundred and ten presumed *E. coli* isolates were identified to the species level by using API^®^ biochemical test strips. From 51 of the 54 positive samples, we identified multiple isolates (2–4), while one isolate per sample was identified in all remaining samples (see Supplementary Table 1). Six phylogroups were detected among the 110 isolates, and in many cases, *E. coli* isolates with different phylogroups occurred in the same sample. For instance, three phylogroups were detected in sample 99 from a broiler barn. **Table 3** shows the number of typed isolates from poultry and pig barns assigned to each phylogenetic group. Phylogroups A and B1 dominated regardless of species. Isolates assigned to groups B2, C, D, and E originated from broiler barns only. When groups A and B1 were compared to each other in terms of isolation media, B1 isolates were more frequently obtained from MacCCIP (chi-square test, *p* = 0.0026). This indicates that reduced susceptibility to CIP was more frequent in phylogroup B1.

**Table 3:** Number of *Escherichia coli* isolates in each phylogenetic group.

Isolate Source	Phylogroup A	Phylogroup B1	Phylogroup B2	Phylogroup C	Phylogroup D	Phylogroup E
Poultry barns	32	39	1	2	6	4
Pig barns	16	10	0	0	0	0

### Antimicrobial Susceptibility Using a Microdilution Test

All typed *E. coli* isolates (*n* = 110) were tested for antimicrobial susceptibility to 10 antibiotics. Results are shown in **Table 4** and include isolation media. Resistance to FIX, AMP, and TET occurred most frequently, then resistance to CHL, SXT, and CIP. Almost one-third of all isolates were resistant to AUG2. Six isolates were resistant to GEN, three to FOX, and all isolates were susceptible to AXO. The difference between resistance rates of isolates from MacC and MacCCIP were below 20%, with the exception of SXT (31%) and CIP (59%).

**Table 4:** Frequencies of phenotypic antibiotic resistance detected in *Escherichia coli* isolates from MacConkey agar with and without ciprofloxacin supplementation.

	AUG2	AMP	FOX	AXO	CHL	CIP	GEN	FIS	TET	SXT
Number (%) of resistant isolates from MacC	18 (23)	55 (70)	1 (1)	0 (0)	42 (53)	28 (35)	4 (5)	79 (100)	54 (68)	36 (46)
Number (%) of resistant isolates from MacCCip	13 (42)	24 (77)	2 (6)	0 (0)	21 (68)	29 (94)	2 (6)	30 (97)	26 (84)	24 (77)
Total no. (%) of resistant isolates	31 (28)	79 (72)	3 (3)	0 (0)	63 (57)	57 (52)	6 (5)	109 (99)	80 (73)	60 (55)

*AUG2, amoxicillin/clavulanic acid; AMP, ampicillin; FOX, cefoxitin; AXO, ceftriaxone; CHL, chloramphenicol; CIP, ciprofloxacin; GEN, gentamicin; FIS, sulfisoxazole; TET, tetracycline; SXT, trimethoprim/sulfamethoxazole; MacC, MacConkey agar without CIP; MacCCip, MacConkey agar with 2 μg/ml CIP.*

**FIGURE 2:**
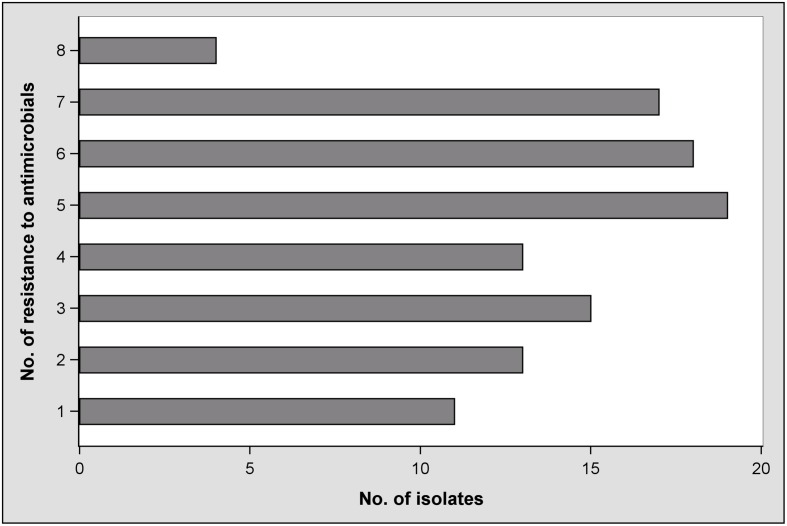
**Frequencies of antimicrobial resistance in *Escherichia coli* isolates**.

Furthermore, the number of antibiotics that individual isolates were resistant to was as high as eight. **Figure 2** illustrates the frequencies of the number of antibiotics isolates were resistant to. The distribution was more or less equal with the exception of the highest number, or eight antibiotics. Only 11% (*n* = 11) of all isolates were resistant to only one antibiotic. More than half (*n* = 58) were phenotypically resistant to five or more antibiotics. However, the number of antibiotics might have been influenced by factors such as the age or origin of the sample. Such coherences were evaluated.

### Effects on the Number of Resistances in *E. coli* Isolates: Results of a Linear Mixed Model

Four selected factors were evaluated for their effects on the number of antibiotics that *E. coli* isolates were resistant to. **Table 5** shows that two factors, isolate age and animal group, had significant effects on the number. Isolate age was negatively correlated (i.e., older isolates were resistant to fewer antibiotics). To compare animal groups, the differences of least squares means was used, and are shown in **Table 6**. With an estimated difference of 3.25, a significant result can be observed for the comparison between isolates from fattening poultry and laying hens, indicating that dusts collected from fattening poultry barns yielded isolates that were resistant to more antibiotics. No significant differences were found between isolates from fattening poultry and pigs or pigs and laying hens, respectively.

**Table 5:** Results of the multi-factorial model used to determine associations with the number of antibiotic resistances.

Fixed effect	Level^∗∗^	Mean	95% Confidence interval	Estimate^∗^	*p*-value
Medium					0.0640
	0	3.6576	2.7439; 4.5713		
	1	4.4487	3.3215; 5.5758		
Animal group					0.0490
	Fattening poultry	5.6723	4.3412; 7.0033		
	Laying hens	2.4213	0.0965; 4.7460		
	Pigs	4.0659	2.4117; 5.7201		
Age of isolates (months)				-0.0180	0.0097
Phylogroup					0.0619
	A	3.6854	2.7022; 4.6685		
	B1	4.6042	3.7531; 5.4553		
	D	3.8698	2.2516; 5.4881		

*^∗^Estimated regression parameter for continuous fixed effects. ^∗∗^0 = MacConkey agar without ciprofloxacin, 1 = MacConkey agar with ciprofloxacin.*

**Table 6:** Differences between least square means of the significant fixed effect “animal group.”

Group 1	Group 2	Estimate	*p*-value
Fattening poultry	Laying hens	3.2510	0.0219
Fattening poultry	Pigs	1.6064	0.0954
Laying hens	Pigs	-1.6446	0.1819

## Discussion

This is the first study to report the long-term viability of *E. coli* in dust samples collected from pig and poultry barns. It reveals that *E. coli* can survive in stored sedimentation dust samples from pig and poultry barns for more than 20 years. It is non-controversial that frozen *E. coli* in pure cultures can survive for decades (Koenig, 2003). There is also evidence that *E. coli* cultures can survive for more than a decade on slants at room temperature (Karcher, 1995). In contrast, survival of this bacterium in secondary habitats is affected by several factors, and survival times of nearly 1 year were estimated (van Elsas et al., 2011). Proliferation of *E. coli* at a storage temperature of 4°C and in material with low water content and low water activities can be widely ruled out (Presser et al., 1998). Even xerophile spore formers cannot grow when *a*_w_ is below 0.65 (Ponizovskaya et al., 2011). We found that the mean *a*_w_ was below 0.6 among our samples. It can be assumed that there was a loss of cultivable aerobic mesophilic bacteria during storage. For instance, the medians of the total viable counts were lowest in samples collected from 1980 to 1984 and those collected from 1985 to 1989 (**Figure 1**). The viability of *E. coli* might have been also affected. This would explain why *E. coli* was not detectable in samples from these time periods and that the detection frequency increased in later time periods.

*Escherichia coli* was found in samples collected as early as 1994 and was detected in each of the time periods after 1994. However, *E. coli* concentrations did not correlate with sample age. The same applies for the concentrations of total viable counts. Although the reason for this remains unknown, it should be considered that the original concentrations of aerobic mesophilic bacteria and of *E. coli* in the dust may have differed by several factors of 10 before storage (Hartung and Saleh, 2007).

*Escherichia coli* survived surprisingly long in dust samples. This might be explained by the route that *E. coli* takes, from animals’ intestines to their feces to airborne particles that accumulate in sedimentation dust, which represents a stepwise loss of water. This process allows adaption to dryness, which is a fundamental advantage for long-term survival of stored *E. coli* (Louis et al., 1994). This agrees with estimates made by others that slow drying of *E. coli* increases its resistance to water loss (Potts, 1994). A consequence of inhibited growth and metabolism in the stored dust samples could be inactivation of autolytic enzymes and of the autolysis process (Lewis, 2000). Furthermore, desiccated bacteria at low water activities are significantly less susceptible to reactive oxygen species (Kim et al., 1999). Oxygen was present when the samples were sealed, and transmission through the used corks cannot be ruled out. However, storage conditions and possibly dust content, such as proteins, may have protected cells from significant effects. Alongside the adaption to dryness and the storage in relatively dry material, the storage temperature is very likely another important factor that influences the long-term survival of *E. coli*. Storage at 4°C seems to be a favorable condition for the survival of *E. coli* (Flint, 1987; Sampson et al., 2006). This may be because of the stability of essential cell structures at this temperature. However, this is only an assumption because the resistance of such structures in viable bacteria cells under dry and cold (unfrozen) conditions is poorly understood.

*Escherichia coli* that survived in sedimentation dust belonged primarily to phylogenetic groups A and B1. Isolates of these groups belong to the commensal microbiota of farm animals and were frequently found on floors of pig and poultry farms in a previous study (Cortes et al., 2010). *E. coli* of phylogenetic group B2, and to a lesser extent of group D, are potential extraintestinal pathogenic bacteria, and they were rarely found in the stored dust. Nevertheless, isolates of these phylogroups survived more than 10 years. This indicates that dust from animal farms is a carrier of potential pathogenic *E. coli*, and emissions or entrainment via contaminated surfaces may lead to transmission into the environment or to other herds. When the most prevalent phylogroups found in this study were compared, significantly more isolates from MacCCIP belonged to group B1. The reason for this remains unknown but may be associated with distinct recombination events in different phylogroups (Wirth et al., 2006).

The earliest resistant isolates were obtained from samples collected in 1994. Unfortunately, no *E. coli* isolates could be cultivated from samples taken between 1980 and 1989. Thus, we are unable to estimate if CIP-resistant *E. coli* were present on farms before fluoroquinolones were introduced into the European market for use in animal husbandries. Fluoroquinolone resistance is common in *E. coli*, and use of the drugs in human and veterinary medicine can increase the number of resistant isolates (van den Bogaard et al., 2001; Redgrave et al., 2014). The relative frequency of all CIP-resistant *E. coli* isolates in this study was 52%. Compared to other studies that cultivated isolates from farm animals (Guerra et al., 2003; Kaesbohrer et al., 2012), we found a higher frequency of resistant isolates. However, it must be considered that the dust samples are not a representative collection of samples and that the partial isolation from MacCCIP is a pre-selection step that affects results. For instance, 94% of the isolates from MacConkey agar with CIP showed phenotypic resistance to CIP in the microdilution test.

In contrast, 35% from MacC showed phenotypic resistance. Ciprofloxacin-resistant *E. coli* occurred in dust samples collected from pig and poultry barns during different time periods. This might be associated with the antibiotic regimen followed within each barn, and this must be carefully discussed. For instance, enrofloxacin is the commonly used fluoroquinolone in animal production that leads to a selection of resistant *E. coli* after oral application (Scherz et al., 2014). Because CIP and enrofloxacin are completely cross-resistant (van den Bogaard et al., 2001), CIP-resistant *E. coli* might occur after animals are treated. On the other hand, once *E. coli* becomes resistant to fluoroquinolones, it will maintain chromosomal mutations and resistance without selective pressure (Freye and Jackson, 2013). Therefore, CIP-resistant *E. coli* might have been shed in detectable amounts by untreated animals.

A retrospective view on the antimicrobial susceptibilities of our *E. coli* isolates reveals that they were resistant to up to eight of 10 antibiotics tested (**Figure 2**). The plate format used for the microdilution test was chosen because it delivers comparable results to antimicrobial drug resistance in *E. coli* isolates whether they are from human or animal sources (Tadesse et al., 2012), and it enabled testing for CIP resistance. Fifty-eight isolates showed resistance to five or more antibiotics. Fifty-three percent were resistant to at least three of the eight drug classes included. According to the definition by Freye and Jackson (2013), these isolates would be classified as multidrug resistant. Resistance to FIS was predominant and observed in 99% of the isolates. It is known that sulfonamide resistance genes are frequently located on plasmids and integrons and that these mobile elements are widespread among *E. coli* from food animals (Freye and Jackson, 2013). If this is also the case in our study, it can be clarified in future studies using additional molecular techniques. Compared to AUG2, FOX, AXO, and GEN, resistance to TET, AMP, SXT, and CHL is clearly greater (**Table 4**). Because antibiotic usage in the sampled barns is unknown, it would be speculative to discuss the differences. However, with the exception of resistance to CHL, our findings are similar to what is deemed “a common multiresistance pattern” distributed and maintained in animal husbandries (Szmolka and Nagy, 2013).

Chloramphenicol-resistant isolates were found in dust samples from all time periods even though the use of this antibiotic in animal production has been prohibited since 1994 in the European Union. It is not clear what led to the remaining resistance without the selective pressure of the antibiotic. Mechanisms leading to multiple resistance or cross-resistance must be taken into account (Speer et al., 1992) and could have been responsible for the relatively high amount of chloramphenicol-resistant isolates.

It is known that antibiotic resistance may be lost in stored bacteria (Breese and Sharp, 1980) and that the number of antibiotic resistances in *E. coli* can vary significantly between isolates from various farm animal species (van den Bogaard et al., 2001). Additionally, own results revealed that the type of isolation medium affected the detection of phylogenetic groups. Therefore, sample origin (pig barn, fattening poultry barn, or laying hen house), age of isolates, type of medium, and phylogenetic group were the variables used in a model to determine whether these factors are associated with the number of antibiotic resistances. Results revealed two significant effects. One was the animal group from which the isolates originated. *E. coli* from fattening poultry barns showed significantly higher numbers of phenotypic resistances to antibiotics than isolates from laying hen flocks. van den Bogaard et al. (2001) and Kaesbohrer et al. (2012) showed, for instance, lower resistance rates and lower prevalences of resistant *E. coli* from laying hens than from fattening poultry. The analyses of isolates from dust collected from such production systems and use of the model reflects a similar trend, although the antimicrobial substances used for detecting resistances differed, in part, from those used in other studies. It is possible that more antibiotics were used in barns with fattening poultry. Furthermore, older isolates showed fewer resistances to the tested antibiotics than younger isolates. It is not known whether this was a consequence of more resistant types spreading in the sampled barns in later time periods. However, other biological reasons should be taken into account. For example, *E. coli* types with fewer resistances may persist longer in dust samples than those with more resistances. Also, it cannot be ruled out that phenotypic resistance was lost by losing genes or by mutations. Perhaps a loss of plasmids during long-term storage and mutations through oxidation, which can accumulate in stored isolates when repair systems cannot work (Zhao and Winkler, 2000; Marston et al., 2005), play a part. We suggest performing targeted sequence analyses in future studies to clarify whether phenotypic resistances were lost during long-term storage. Furthermore, sequencing of the entire genome, for instance by next-generation sequencing (NGS), may deliver information about the alteration of DNA during storage of unfrozen bacteria cells under dry conditions. Can such cells accumulate mutations? Dry, relatively cold, and dark habitats exist also in nature, and it would be interesting to investigate whether *E. coli* accumulates mutations and phenotypical changes under such conditions.

## Author Contributions

JS planned the study, identified the isolates, conducted the antimicrobial susceptibility testing, and carried out the data analysis and basic statistics. IR programmed a linear mixed model, analyzed the results of the model, and wrote the sections “Statistical Analyses” and “Effects on the Number of Resistances in *E. coli* Isolates: Results of a Linear Mixed Model.” JH sampled the dust, stored the dust samples, and wrote the section “Origins of Dust Samples.” CE performed the phylotyping, analyzed the results, and wrote parts of sections “Material and Methods” and “Results.” GH measured the water activities and wrote the section “Water Activities of Dust Samples” from both “Materials and Methods” and “Results.” JS and NK wrote the manuscript. All authors read and approved the final manuscript.

## Conflict of Interest Statement

The authors declare that the research was conducted in the absence of any commercial or financial relationships that could be construed as a potential conflict of interest.
